# Developmental Origins of Pregnancy-Related Morbidity and Mortality in Black U.S. Women

**DOI:** 10.3389/fpubh.2022.853018

**Published:** 2022-06-13

**Authors:** Betty Lin, Allison A. Appleton

**Affiliations:** ^1^Department of Psychology, College of Arts and Sciences, University at Albany, State University of New York, Albany, NY, United States; ^2^Department of Epidemiology and Biostatistics, School of Public Health, University at Albany, State University of New York, Rensselaer, NY, United States

**Keywords:** pregnancy-related morbidity and mortality, health disparities, Black women, women's health, race, intergenerational transmission, developmental origins of adult disease

## Abstract

In the US, Black women are at disproportionate risk for pregnancy-related morbidity and mortality (PRMM). Disparities in PRMM have been tied to elevated rates of obstetric cardiometabolic complications for Black women. Research seeking to elucidate the determinants of Black PRMM to date have focused predominantly on risk factors occurring during pregnancy (e.g., health risk behaviors, quantity and quality of prenatal care, provider behaviors, and attitudes). Meanwhile, other research investigating the developmental origins of health and disease (DOHaD) model indicates that the origins of adult cardiometabolic health can be traced back to stress exposures occurring during the intrauterine and early life periods. Despite the relevancy of this work to Black PRMM, the DOHaD model has never been applied to investigate the determinants of Black PRMM. We argue that the DOHaD model represents a compelling theoretical framework from which to conceptualize factors that drive racial disparities PRMM. Research and intervention working from a developmental origins orientation may help address this urgent public health crisis of Black PRMM.

## Introduction

Although the vast majority of Black American women experience healthy pregnancies, Black women continue to experience unacceptably high rates of pregnancy-related morbidity and mortality [PRMM; (1)]. Black American women are 1.5–4 times more likely to die within a year of birth compared to other racial/ethnic groups (2). Rates of PRMM represent the “tip of the iceberg,” and are linked to even greater numbers of women suffering from related health concerns with implications for longevity and wellbeing (3). Furthermore, elevated rates of Black PRMM contribute to disparities in Black infant birth and health outcomes (4). Compared to their non-Black counterparts, Black infants are 2–3 times more likely to be born preterm or low birthweight or to die within a year of birth (4). The root causes of Black PRMM are unknown, yet cardiometabolic complications (e.g., hypertension, cardiomyopathy, preeclampsia, gestational diabetes) are known to play a role in a majority of cases (5). The fact that cardiometabolic complications represent a key risk mechanism underlying disparities in PRMM is salient given that Black Americans also experience disproportionate risk for developing cardiometabolic health conditions in mid- to late-life more generally, and begs the consideration that health disparities in PRMM and cardiometabolic health may share common origins.

Nevertheless, efforts to identify the determinants of these disparities to date have focused predominantly on risk factors occurring during pregnancy, specifically at the patient (e.g., sociodemographic, health knowledge and behaviors, social supports), provider (e.g., experience, cultural competence, communication), and system (e.g., hospital quality, health policy) levels (6). This work has afforded important insights about contemporaneous factors that may exacerbate risk for PRMM, and contributes importantly to conversations about possible proximal targets for screening and intervention. However, insights from other research suggest that the origins of risk for PRMM likely far predate pregnancy. In particular, research investigating the Developmental Origins of Health and Disease (DOHaD) model has now yielded robust evidence that the foundations of adult health and disease—including cardiometabolic health—can be traced back to environmental exposures occurring during the fetal and early life years. Despite the relevancy of this work for PRMM, this model has not yet been applied to understand determinants of PRMM or racial disparities therein. We discuss the utility of the DOHaD model for identifying factors that drive Black PRMM. Elucidating the developmental origins of Black PRMM and counteractive resiliency factors is critically needed to address this urgent public health crisis.

## The Developmental Origins of Health and Disease Model

The DOHaD model was first proposed to explain unexpectedly high rates of adult cardiovascular disease in geographic regions that were otherwise enjoying increasing prosperity [e.g., post-war Britain from the 1950s to 1980s; (7)]. Specifically, given that rates of cardiovascular disease were known to correspond with socioeconomic status (SES), the fact that they were rising steeply in regions enjoying improving social and economic conditions was paradoxical. To investigate the reasons for this paradox, Barker (7) launched a series of studies which revealed that contemporaneous geographic distributions of cardiovascular disease paralleled *past* geographic distributions of infant mortality. These and related observations led Barker and colleagues to hypothesize that the same factors that contributed to risk for infant poor birth outcomes and mortality also contributed latent risk for poor cardiovascular health that would manifest years later in adulthood. This hypothesis ultimately served the basis of what would become the DOHaD model, which posits that the foundations of adult health and disease are “programmed,” or shaped, by prenatal environmental exposures (7).

Since its inception, research evaluating the DOHaD model has demonstrated robust links between intrauterine exposures such as maternal undernutrition, which is prominent in impoverished regions, and adult cardiometabolic health (8). Within the DOHaD literature, the prenatal environment refers to the intrauterine milieu, and prenatal stress is defined as alterations to the intrauterine milieu that disrupt homeostasis. For clarity, we refer to each as the “intrauterine environment” and “intrauterine stress” throughout. Mechanistically, intrauterine stress is thought to prompt enduring neurobiological, metabolic, and epigenetic alterations with implications for adult health and disease [see (9, 10) for more detailed discussions]. These alterations are thought to promote offspring adaptation in environments consistent with the intrauterine environment (e.g., food scarcity via maternal undernutrition), but potentiate risk for the development of cardiometabolic disorders later in life. For some, these alterations can elicit homeostatic adjustments to fetal growth that can manifest as preterm birth, low birthweight, and intrauterine growth restriction—side effects of programming processes that account for the correspondence of historical birth outcomes with future cardiometabolic health (11). The DOHaD model initially focused on the contributions of maternal undernutrition to risk for adult cardiometabolic disease, but has since been expanded to encompass the contributions of a broader range of intrauterine and early life stressors ranging from psychosocial to chemical (12).

## Paradoxical Findings About SES and Black PRMM

The prominent and sometimes unexpected roles socioeconomic status, women's cardiometabolic conditions, and infant birth outcomes played in early DOHaD research bear striking parallels to findings from research investigating Black PRMM to date. In fact, one of the most puzzling findings about disparities in Black PRMM is that they are most pronounced among Black women with high SES (5, 13). Specifically, while Black women with high SES have comparably better pregnancy-related outcomes compared to Black women with low SES (14), Black women with high SES are at considerably greater risk for poor pregnancy-related outcomes compared to White women with high *or* low SES (13, 15). Drawing data from the Center for Disease Control's Pregnancy Mortality Surveillance System from 2007 to 2016, Petersen et al. (5) reported that college-educated Black women were 5.2 times more likely to die from pregnancy-related complications within a year of pregnancy than college-educated White women, and were 1.6 times more likely to die than White women with *less than a high school education*. These statistics have been personified by high-profile cases that have appeared in popular media, in which prominent Black women including Beyonce Knowles-Carter and Serena Williams—both of whom have exceptionally high social *and* economic standing—have shared about their own experiences with life-threatening pregnancy-related cardiometabolic complications (16, 17). Thus, while high SES at the time of pregnancy affords Black women some protection from PRMM, Black women with high SES are still at greater risk for PRMM than White women with low SES.

Whereas, high SES *at the time of pregnancy* does not appear to explain disparities in Black PRMM, women's mother's SES when women themselves were *in-utero* (henceforth “maternal intrauterine SES,” MI-SES) may. Few or no studies have explicitly examined the impact of MI-SES on PRMM. However, emergent research has demonstrated links between MI-SES and later birth outcomes—the latter which again often co-occurs with PRMM. Indeed, Black women's *own* intrauterine exposure to neighborhood deprivation was found to predict their infant birth outcomes independent of contemporaneous neighborhood deprivation (18). Likewise, MI-SES (i.e., women's mothers' education during pregnancy) has been associated with infant birth outcomes over and above the effects of women's pregnancy SES [i.e., educational attainment; (19)]. Thus, and in parallel to early observations by Barker and colleagues that distributions of cardiovascular disease were more closely associated with historical than contemporaneous prosperity, Black women's MI-SES-linked stress exposures may better explain PRMM than socioeconomic exposures at the time of pregnancy.

**Figure 1:**
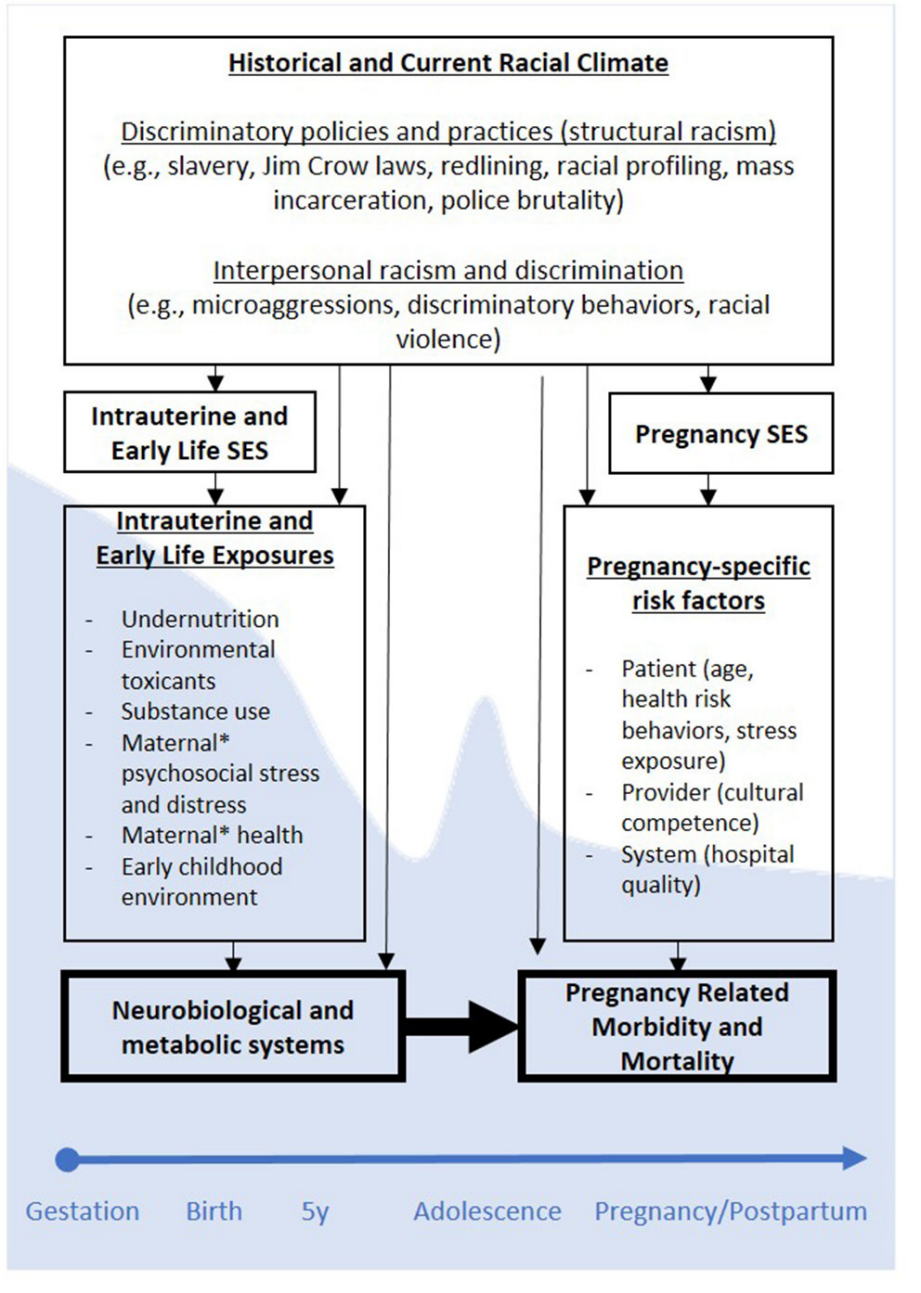
Conceptual Model of Developmental Origins of Black PRMM. *Maternal refers to women's biological mothers. SES, socioeconomic status; PRMM, pregnancy-related mortality and morbidity. Programming effects as hypothesized using a DOHaD framework are depicted across one generation, but may be cyclical across generations (e.g., generation 1 PRMM may contribute to programming of generation 2 neurobiological and metabolic systems, etc.). Shaded (blue) backdrop corresponding to different developmental periods indicates neuroplasticity, and degrees to which stress exposures during those periods may prompt programming processes.

Viewed from a DOHaD framework, low MI-SES may heighten risk for PRMM by prompting the developmental programming of foundational neurobiological and metabolic systems that underlie adult health, including reproductive health (see Figure 1). If programming effects secondary to such stress exposures indeed contribute to PRMM, then low MI-SES may help explain why high SES Black women—many who themselves were born into more impoverished conditions (20, 21)—continue to experience elevated risk for PRMM despite current high SES. Stated differently, MI-SES may program latent health risk for PRMM irrespective of high SES at the time of pregnancy. Consistent with this suggestion, a study that drew data from the Illinois transgenerational birth file yielded findings that although Black women with upward socioeconomic mobility (i.e., higher SES than *their* mothers when women were *in-utero*) had better pregnancy outcomes than Black women without, these benefits did not extend to women who themselves were born low birthweight (14). Remembering again that low birthweight is thought to represent an indicator of intrauterine programming processes (11), these findings align with the DOHaD model, and suggest that health advantages associated with upward economic mobility are more limited for Black women who themselves were impacted by developmental programming *in-utero*. In sum, compelling evidence suggests MI-SES may account for racial disparities in Black PRMM in a manner consistent with the DOHaD model. Efforts to integrate insights from DOHaD may prove fruitful for elucidating the mechanisms through which health disparities are perpetuated across generations.

## Characterizing Stress Exposures Among Black Women

Although early DOHaD research focused primarily on intrauterine exposure to maternal undernutrition as a contributor to adult health, maternal undernutrition is now known to represent only one of several possible stress exposures that can heighten risk for poor cardiometabolic health. Research has additionally revealed that exposures that may prompt programming processes likely extend beyond the intrauterine exposures to also include early life exposures—particularly during periods of marked neuroplasticity (e.g., early childhood, adolescence). Accordingly, intrauterine and early life stress exposures that can prompt developmental programming processes and occur during sensitive developmental periods represent prime targets in need of investigation as potential determinants of Black PRMM. We focus discussion on stress exposures that may prompt developmental programming consistent with the DOHaD model, but urge scholars to simultaneously consider the resiliency factors that contribute to the healthy pregnancies most Black women experience.

### Socioeconomically-Linked Exposures

In addition to maternal undernutrition, exposure to SES-linked intrauterine and early life stressors including poor maternal health (i.e., women's mothers' health conditions, pregnancy complications), environmental toxicants (pollutants, heavy metals), drugs and substances (including over the counter drugs, prescription medications, tobacco), maternal psychosocial stress and distress, and childhood trauma and adversity have all been implicated in programming processes underlying cardiometabolic health (12, 22). As we alluded to previously, many of these SES-linked exposures have been raised as potential risk factors underlying PRMM. However, whereas these conceptualizations have focused on links between contemporaneous SES-linked exposures with PRMM, insights from the DOHaD framework urge a need to consider the role of pre-conception (e.g., intrauterine and early life) SES-linked exposures with PRMM, the latter which may have programmed latent risk for adult cardiometabolic health, and correspondingly, PRMM well before pregnancy.

Critically, SES-related exposures extend beyond the individual level to also include neighborhood- and community-level exposures. On average, low SES neighborhoods tend to be fraught with material and psychosocial risk factors that may heighten risk for intrauterine or early life exposure to each of the aforementioned stressors, including decreased access to quality and affordable food, health care, and education; decreased opportunities for recreation and physical activity; increased exposure to environmental contaminants, and lower neighborhood safety (23). In fact, the sum of these and related indices of neighborhood quality have been found to correspond with healthier pregnancy outcomes (24), even when controlling for family-level SES (25). As such, efforts to delineate the origins of risk for PRMM must also consider neighborhood- and community-level systems and structures that contribute to risk for PRMM over and above immediate impacts on family SES.

### Sociopolitical Determinants

Socioeconomic and residential inequalities are further tied to policies and practices—past and present—that have helped to generate and maintain structural inequality. First, it is notable that the Civil Rights Acts of 1964 and 1968—which made racial economic and residential discrimination illegal—were only codified into law ~50 years ago. Prior to this time, practices such as “redlining”—the federally-sponsored practice of delineating neighborhoods as undesirable based on factors including racial composition—systematically impeded capacities for Black Americans to achieve upward economic mobility while also contributing to the worsening conditions in majority Black neighborhoods (26). Second, even after racial discrimination became illegal, decades of discrimination and segregation had already laid the groundwork for a social and economic landscape rife with inequality. Third, although explicitly discriminatory practices are now outlawed, more subversive forms thereof continue to perpetuate and maintain existing inequalities (27). Indeed, historically redlined neighborhoods have largely remained economically impoverished and racially segregated (26), and correspondingly are burdened by many of the aforementioned SES-related health risks (28).

Accordingly, women living in historically redlined neighborhoods have been found to have poorer pregnancy-related outcomes (i.e., low birthweight) than those living in historically desirable neighborhoods even when controlling for women's own sociodemographic factors and current census tract-level poverty (25). The legacies of these and other health-impeding policies and practices represent a critical backdrop within which to contextualize socioeconomic and health disparities in Black Americans. Moreover, considering that (1) the Civil Rights Acts were only passed ~2–3 generations ago when most contemporary grandmothers and great grandmothers themselves were *in-utero*—and (2) many of the stress exposures tied to these policies and practices are cyclical across generations (e.g., maternal pregnancy health status in one generation can program intergenerational risk for pregnancy health status in the subsequent generation), the DOHaD model elucidates a mechanism through which the health implications of racist practices and policies can reverberate across generations. The DOHaD model additionally suggests that, without intervention, risk for PRMM may likely be compounded across generations. Thus, efforts to understand and address disparities in Black PRMM and corresponding birth outcomes may be even more urgent than currently appreciated.

### Race-Related Psychosocial Determinants

The determinants of Black PRMM likely extend beyond the transgenerational impact of low SES or poor neighborhood conditions alone. In fact, considering the unique historical *and* current events that have shaped the experience of being a Black American today, the breadth of offending stressors is likely more complex, nuanced, and expansive than has been considered in DOHaD research to date. Consistent with this suggestion, lifetime SES has been found to account for a smaller share of variance in pregnancy-related health outcomes for Black compared to White women (15). Additionally, risk for adverse pregnancy-related outcomes has been found to increase as a function of generational status, and to emerge only among Black women who were born in or immigrated early to the US (29). Thus, risk is more closely tied to social rather than genetic race-related determinants. Concordantly, growing scholarship has underscored the importance of considering the health impacts of race-related stressors as well as of stressors lying at the intersections of race and gender (30). These stressors may take various forms, including teasing, bullying, explicitly discriminatory interactions, microaggressions, and vicarious discrimination (31, 32)—all of which may further contribute to general feelings of unsafety (33). Race-related psychosocial stressors are prominent across SES strata, yet may be particularly prominent for Black women and girls with high SES who also spend more time in settings in which they are underrepresented (34).

Congruently, Black women with high SES have been found to have the best birth outcomes when living in predominantly Black neighborhoods, but the worst birth outcomes when living in predominantly White neighborhoods (35). Similarly, Black women with positive income incongruity (i.e., living in wealthier neighborhoods than other Black women with comparable SES) have been found to have healthier pregnancy outcomes when living in predominantly Black vs. racially mixed neighborhoods (36). To be clear, we do not take these findings to be indicative of health benefits of racial segregation, rather to reflect longer-standing problems associated with limited intergroup contact (e.g., more prejudicial attitudes and behaviors) that have arisen *because of* racial segregation. Instead, we raise these issues to highlight a need for research to investigate the extent to which experiences of interpersonal racism and discrimination may directly or interactively undermine Black women's health. Furthermore, in light of evidence suggestive of potential health benefits secondary to residence within predominantly Black communities, efforts to delineate the cultural and community resiliency factors that promote health are equally critical.

## Discussion

In sum, rates of Black PRMM remain unacceptably high, and efforts to elucidate and address the root causes of these disparities is urgently needed to address this public health crisis. Important insights have been gleaned from extant research evaluating individual, provider, and system-level factors that exacerbate risk for Black PRMM, yet this work has lacked an organizing theoretical framework from which to conceptualize the origins of Black PRMM. Striking parallels have emerged between research investigating DOHaD and Black PRMM, and compelling evidence suggests that the origins of Black PRMM can be traced back to women's intrauterine and early life exposures. Thus, efforts to integrate insights afforded by extant and forthcoming research within a DOHaD framework may help to propel research investigating the determinants of Black PRMM.

As we have started to delineate, the stressors that contribute to Black PRMM are likely multifactorial, intergenerational, and involve the unique interplay of multi-level stressors. Additionally, multiple risk trajectories likely contribute to disparities in Black PRMM. Greater attention to stress exposures prominent spanning each of the putative sensitive developmental periods (i.e., intrauterine, infancy and early childhood, adolescence) may help to clarify the degree to which such exposures may exert additive, multiplicative, or re-programming effects on cardiometabolic health with significant implications for intervention. Taken together, this research urges a need for researchers, practitioners, and policymakers to consider a broader array of determinants across development than considered to date. Such efforts will likely warrant a need for multidisciplinary investigations that can leverage insights and methods from varied disciplines. In the interim, while research continues to investigate determinants of Black PRMM, it may be fruitful for practitioners to consider screening for indicators of intrauterine programming (e.g., birthweight, preterm birth) to help identify women with risk for PRMM.

Importantly, culturally responsive applications of the DOHaD framework will necessarily contextualize stress investigations within the unique historical and current events that have shaped and continue to shape generations of Black American lives. Finally, although we have focused our discussion on risk processes consistent with the DOHaD model, we cannot emphasize enough that the vast majority of Black women experience healthy pregnancies despite these risks. Thus, efforts to delineate the myriad individual, family, community, and cultural strengths that disrupt or counteract putative intrauterine and early life programming processes represent equally important targets for future research.

## Data Availability Statement

The original contributions presented in the study are included in the article/supplementary material, further inquiries can be directed to the corresponding author/s.

## Author Contributions

BL and AA conceived the manuscript and edited and revised subsequent drafts. BL drafted the initial manuscript draft. Both authors contributed to the article and approved the submitted version.

## Funding

This research was supported by funds provided by the State University of New York Multidisciplinary Small Team Grant (RSG201024.2).

## Conflict of Interest

The authors declare that the research was conducted in the absence of any commercial or financial relationships that could be construed as a potential conflict of interest.

## Publisher's Note

All claims expressed in this article are solely those of the authors and do not necessarily represent those of their affiliated organizations, or those of the publisher, the editors and the reviewers. Any product that may be evaluated in this article, or claim that may be made by its manufacturer, is not guaranteed or endorsed by the publisher.

## References

[1] Alkema L Chou D Hogan D Zhang S Moller AB Gemmill A. Global, regional, and national levels and trends in maternal mortality between 1990 and 2015, with scenario-based projections to 2030: a systematic analysis by the UN Maternal Mortality Estimation Inter-Agency Group. Lancet. (2016) 387:462–74. 10.1016/S0140-6736(15)00838-7 26584737 PMC5515236

[2] Singh GK. Trends and social inequalities in maternal mortality in the United States, 1969-2018. Int. J. MCH AIDS. (2021) 10:29–42. 10.21106/ijma.444 33442490 PMC7792749

[3] Firoz T Chou D von Dadelszen P Agrawal P Vanderkruik R Tunçalp O. Measuring maternal health: focus on maternal morbidity. Bull World Health Organ. (2013) 91:794–6. 10.2471/BLT.13.117564 24115804 PMC3791656

[4] Singh GK Yu SM. Infant mortality in the United States, 1915-2017: large social inequalities have persisted for over a century. Int J MCH AIDS. (2019) 8:19–31. 10.21106/ijma.271 31049261 PMC6487507

[5] Petersen EE Davis NL Goodman D Cox S Syverson C Seed K. Racial/Ethnic disparities in pregnancy-related deaths - United States, 2007-2016. MMWR Morb Mortal Wkly Rep. (2019) 68:762–5. 10.15585/mmwr.mm6835a3 31487273 PMC6730892

[6] Howell EA. Reducing disparities in severe maternal morbidity and mortality. Clin Obstet Gynecol. (2018) 61:387–99. 10.1097/GRF.0000000000000349 29346121 PMC5915910

[7] Barker DJ. The origins of the developmental origins theory. J Intern Med. (2007) 261:412–7. 10.1111/j.1365-2796.2007.01809.x 17444880

[8] Rich-Edwards JW Stampfer MJ Manson JE Rosner B Hankinson SE Colditz GA. Birth weight and risk of cardiovascular disease in a cohort of women followed up since 1976. BMJ. (1997) 315:396–400. 10.1136/bmj.315.7105.396 9277603 PMC2127275

[9] Beijers R Buitelaar JK de Weerth C. Mechanisms underlying the effects of prenatal psychosocial stress on child outcomes: beyond the HPA axis. Eur Child Adolesc Psychiatry. (2014) 23:943–56. 10.1007/s00787-014-0566-3 24875898

[10] Scorza P Duarte CS Hipwell AE Posner J Ortin A Canino G. Research review: intergenerational transmission of disadvantage: epigenetics and parents' childhoods as the first exposure. J Child Psychol Psychiatry. (2018) 60:119–32. 10.1111/jcpp.12877 29473646 PMC6107434

[11] Seckl JR. Glucocorticoid programming of the fetus; adult phenotypes and molecular mechanisms. Mol Cell Endocrinol. (2001) 185:61–71. 10.1016/S0303-7207(01)00633-5 11738795

[12] Yeung EH Robledo C Boghossian N Zhang C Mendola P. Developmental origins of cardiovascular disease. Curr Epidemiol Rep. (2014) 1:9–16. 10.1007/s40471-014-0006-4 25364653 PMC4214855

[13] Ross KM Dunkel Schetter C McLemore MR Chambers BD Paynter RA Baer R. Socioeconomic status, preeclampsia risk and gestational length in black and white women. J Racial Ethn Health Disparities. (2019) 6:1182–91. 10.1007/s40615-019-00619-3 31368002

[14] Collins JW Rankin KM David RJ. African American women's lifetime upward economic mobility and preterm birth: the effect of fetal programming. Am J Public Health. (2011) 101:714–9. 10.2105/AJPH.2010.195024 21330589 PMC3052339

[15] Colen CG Geronimus AT Bound J James SA. Maternal upward socioeconomic mobility and black-white disparities in infant birthweight. Am J Public Health. (2006) 96:2032–9. 10.2105/AJPH.2005.076547 17018818 PMC1751798

[16] Knowles-Carter B. (2018) “Beyoncé in her own words: Her life, her body, her heritage.”, *in:* Vogue.).

[17] Williams S. (2018) *Serena* Williams: What my life-threatening experience taught me about giving birth. CNN. CNN.

[18] Collins JW David RJ Rankin KM Desireddi JR. Transgenerational effect of neighborhood poverty on low birth weight among African Americans in Cook County, Illinois. Am J Epidemiol. (2009) 169:712–7. 10.1093/aje/kwn402 19179359

[19] Kane JB. An integrative model of inter-and intragenerational preconception processes influencing birthweight in the United States. J Health Soc Behav. (2015) 56:246–61. 10.1177/0022146515582043 25953279 PMC4449805

[20] Collins JW Rankin KM David RJ. Low birth weight across generations: the effect of economic environment. Matern Child Health J. (2011) 15:438–45. 10.1007/s10995-010-0603-x 20390329

[21] Pfeffer FT Killewald A. How rigid is the wealth structure and why? Inter-and multigenerational associations in family wealth. Popul Stud Cent Res Rep. No. 15–845. (2015).

[22] Steine IM LeWinn KZ Lisha N Tylavsky F Smith R Bowman M. Maternal exposure to childhood traumatic events, but not multi-domain psychosocial stressors, predict placental corticotrophin releasing hormone across pregnancy. Soc Sci Med. (2020) 266:113461. 10.1016/j.socscimed.2020.113461 33126094 PMC9380779

[23] Evans GW. The environment of childhood poverty. Am Psychol. (2004) 59:77. 10.1037/0003-066X.59.2.77 14992634

[24] Appleton AA Lin B Holdsworth EA Feingold BJ Schell LM. Prenatal exposure to favorable social and environmental neighborhood conditions is associated with healthy pregnancy and infant outcomes. Int J Environ Res Public Health. (2021) 18:6161. 10.3390/ijerph18116161 34200387 PMC8200992

[25] Krieger N Wye GV Huynh M Waterman PD Maduro G Li W. Structural racism, historical redlining, and risk of preterm birth in New York City, 2013–2017. Am J Public Health. (2020) 110:1046–53. 10.2105/AJPH.2020.305656 32437270 PMC7287548

[26] Mitchell B Franco J. “*HOLC “Redlining” Maps: The Persistent Structure of Segregation and Economic Inequality*. (2019). Washington, DC: National Community Reinvestment Coalition.

[27] Quillian L Lee JJ Honoré B. Racial discrimination in the Housing US. and Mortgage lending markets: a quantitative review of trends, 1976–2016. Race Soc Probl. (2020) 12:13–28. 10.1007/s12552-019-09276-x

[28] Smith CL. Economic deprivation and environmental inequality in postindustrial Detroit: a comparison of landfill and superfund site locations. Organ Environ. (2007) 20:25–43. 10.1177/1086026607300245

[29] David R Collins J Jr. Disparities in infant mortality: what's genetics got to do with it? Am J Public Health. (2007) 97:1191–7. 10.2105/AJPH.2005.068387 17538073 PMC1913086

[30] Mehra R Boyd LM Magriples U Kershaw TS Ickovics JR Keene DE. Black pregnant women “Get the Most Judgment”: a qualitative study of the experiences of black women at the intersection of race, gender, and pregnancy. Womens Health Issues. (2020) 30:484–92. 10.1016/j.whi.2020.08.001 32900575 PMC7704604

[31] Sanders-Phillips K Settles-Reaves B Walker D Brownlow J. Social inequality and racial discrimination: risk factors for health disparities in children of color. Pediatrics. (2009) 124:S176–S186. 10.1542/peds.2009-1100E 19861468

[32] Priest N Paradies Y Trenerry B Truong M Karlsen S Kelly Y. A systematic review of studies examining the relationship between reported racism and health and wellbeing for children and young people. Soc Sci Med. (2013) 95:115–27. 10.1016/j.socscimed.2012.11.031 23312306

[33] Brosschot JF Verkuil B Thayer JF. Generalized unsafety theory of stress: unsafe environments and conditions, and the default stress response. Int J Environ Res Public Health. (2018) 15:464. 10.3390/ijerph15030464 29518937 PMC5877009

[34] Braveman P Heck K Egerter S Dominguez TP Rinki C Marchi KS. Worry about racial discrimination: A missing piece of the puzzle of Black-White disparities in preterm birth? PLoS ONE. (2017) 12:e0186151. 10.1371/journal.pone.0186151 29020025 PMC5636124

[35] Kothari CL Paul R Dormitorio B Ospina F James A Lenz D. The interplay of race, socioeconomic status and neighborhood residence upon birth outcomes in a high black infant mortality community. SSM Popul Health. (2016) 2:859–67. 10.1016/j.ssmph.2016.09.011 29349194 PMC5757914

[36] Pickett KE Collins JW Masi CM Wilkinson RG. The effects of racial density and income incongruity on pregnancy outcomes. Soc Sci Med. (2005) 60:2229–38. 10.1016/j.socscimed.2004.10.023 15748671

